# New MEMS Tweezers for the Viscoelastic Characterization of Soft Materials at the Microscale

**DOI:** 10.3390/mi9010015

**Published:** 2017-12-30

**Authors:** Paolo Di Giamberardino, Alvise Bagolini, Pierluigi Bellutti, Imre J. Rudas, Matteo Verotti, Fabio Botta, Nicola P. Belfiore

**Affiliations:** 1 Department of Computer, Control, and Management Engineering Antonio Ruberti, University of Rome La Sapienza, Via Ariosto 25, I-00185 Roma, Italy; paolo.digiamberardino@uniroma1.it; 2 MNF - Micro nano fabrication and characterization Facility, Fondazione Bruno Kessler (FBK), Via Sommarive 18, I-38123 Trento, Italy; bagolini@fbk.eu (A.B.); bellutti@fbk.eu (P.B.); 3 Head of Steering Committee of University Research and Innovation Center, Óbuda University, 96/b Becsi ut, H-1034 Budapest, Hungary; rudas@uni-obuda.hu; 4 Department of Industrial Engineering, University of Trento, via Sommarive, 9-38123 Trento, Italy; matteo.verotti@unitn.it; 5 ProM Facility, Trentino Sviluppo S.p.A., Via Zeni Fortunato, 8, 38068 Rovereto, Italy; 6 Department of Engineering, Universita degli Studi Roma Tre, via della Vasca Navale 79, 00146 Roma, Italy; fabio.botta@uniroma3.it

**Keywords:** MEMS, tweezers, control, viscous coefficient, soft tissue

## Abstract

As many studies show, there is a relation between the tissue’s mechanical characteristics and some specific diseases. Knowing this relationship would help early diagnosis or microsurgery. In this paper, a new method for measuring the viscoelastic properties of soft materials at the microscale is proposed. This approach is based on the adoption of a microsystem whose mechanical structure can be reduced to a compliant four bar linkage where the connecting rod is substituted by the tissue sample. A procedure to identify both stiffness and damping coefficients of the tissue is then applied to the developed hardware. Particularly, stiffness is calculated solving the static equations of the mechanism in a desired configuration, while the damping coefficient is inferred from the dynamic equations, which are written under the hypothesis that the sample tissue is excited by a variable compression force characterized by a suitable wave form. The whole procedure is implemented by making use of a control system.

## 1. Introduction

The manipulation at the micro or nanoscale nowadays mainly regards the handling of small biological tissue samples or cells for a large variety of applications where their specific mechanical characteristics should be known before any possible treatment.

A specialized knowledge about the relations between the tissue sample mechanical characteristics and some specific diseases would help much in diagnosis or microsurgery, but unfortunately it is not easy to develop new experimental tests because of the lack of specialized instrumentation or of its high costs at micro or nanosize. The most typical mechanical characteristic is *stiffness*, although much information is expected to be contained in the *viscoelastic response* to a given exciting stress.

Other than simulations (for example with the aid of Finite Element Analysis) and theoretical work (Continuum Mechanics), many different experimental activities have been attempted in the recent past, such as, for example, stretch, dynamic, aspiration or indentation tests.

Mechanical stretch tests represent the most immediate way to obtain information on a biological material. Since 2000, nonlinear and viscoelastic material properties of brain tissues have been analyzed [1] and their mechanical response has been recognized to be related to strain history. In another investigation [2], mechanical properties of human skin have been characterized through uniaxial tensile testing and histomorphometric analysis. Tension tests showed that pressure ulcer tissues had significantly lower strains (at peak stress) than that characteristic of control healthy breast tissues. The mechanical properties of reconstituted tissues have been also quantitatively characterized by means of uniaxial stretch measurements [3], and the cell density and matrix organization has been studied by analyzing the strain-dependent stress and the elastic modulus of the active component. Multiaxial testing techniques showed [4] how soft biological tissues exhibit complex mechanical behaviors not easily accounted for in classic elastomeric constitutive models. In 2006, quasilinear viscoelasticity and hysteresis in porcine esophagus tissues have been investigated by means of stress-relaxation tests at various strains and Fung’s quasilinear viscoelastic model has been assessed [5]. Incremental relaxation stress tests have been also adopted to identify surface-regional mechanical variations in the porcine temporomandibular joint disc under unconfined compression [6] and a value of the coefficient of viscosity turned out to be approximately equal to 3.5 MPa-s. More recently, some efforts have been paid to characterize tissue-engineered blood vessels by analyzing their mechanical properties by means of compliance measurement, burst pressure testing, creep and cyclic testing. These investigations are helpful to develop efficient fabrication methods and to optimize tissue resistance and viscoelastic properties. For example, the mechanical properties of tissue-engineered vascular constructs have been studied by monitoring pressure and diameter variations of vascular constructs submitted to hydrostatic loading [7]. A piezoelectric device, that measures the properties of the biological tissues, has been also recently developed [8]. The system uses the properties of the piezoelectric materials [9,10,11] to measure the viscoelasticity coefficients of the tissue in the near-surface regions of the epidermis.

Dynamic and vibration based methods offer other means for characterizing materials. For example, in 2004 some dynamic tests have been performed [12] to investigate frequency-dependent mechanical properties of tissue, specifically for properties in compressive, tensile, and flexural modes over a frequency range from 0.1 to 400 Hz. Ultrasound vibrometry has provided noninvasive tools to estimate the shear moduli of tissues from the propagation velocities at multiple frequencies. For example, Voigt, Maxwell and Zener classic models have been applied to estimate shear moduli of porcine livers with different thermal damage levels and different storage times [13].

Some tests have been performed by taking advantage from robotics and control. For example, a haptics-enabled master-slave test-bed has been used [14] to understand how various modalities for feedback of interaction between a surgical tool and a soft tissue can improve the efficiency of a typical surgical task. Furthermore, a multi–axial force sensor mounted on a robotic manipulator, together with a stereo camera system, has been adopted to estimate mechanical parameters of soft tissue from sensory data collected during robotic surgical manipulation [15].

Nanoindentation has been also adopted to characterize nano and microscale mechanical properties in biomaterials [16], although this technique is usually applied to hard tissues such as bone and teeth. In 2008, considering that heart valve tissues show nonlinear, anisotropic and inhomogeneous mechanical behavior, some indentation tests have been performed [17] on linear elastic polydimethylsiloxane rubbers and compared to tensile tests on the same specimen for local characterization of nonlinear soft anisotropic tissue properties. The mechanical relaxation behavior of injured rat spinal cord tissue, at the site of mid-thoracic spinal hemisection, has been monitored post-injury using a microindentation test method in order to calculate the elastic moduli and to estimate the relaxation time constant and viscosity. This activity showed that injured tissues exhibited significantly lower stiffness and elastic modulus in comparison to uninjured control tissue [18]. Results could be used to understand some aspects of biochemical nerve regeneration. Elastic and viscoelastic properties of undifferentiated adipose-derived stem cells have been tested via atomic force microscopy and correlated with lineage-specific metabolite production [19]. Mechanical characterization of soft tissues with atomic force microscopy can be affected by tissue samples viscous effects in case of sharp tips. Hence, different responses to nanoindentation of soft biotissues with different elasticity and viscous properties have been investigated. For example, similarities with indentation of elasto-plastic materials have been ascertained and also a linear relationship between the limit indentation rate and the geometry of the AFM probe has been obtained [20].

In case the probed sample is non-adherent to the rigid substrate, the elastic modulus of cells and biological samples can be evaluated experimentally by means of micropipette aspiration. In 2007, intra-operative aspiration in vivo experiments assessed the mechanical response of human liver [21]. This mechanical characterization was useful to histological evaluation of tissue biopsies to make correlations between mechanical response and tissue micro-structure of normal and diseased liver and various pathologic conditions affecting the tissue samples were quantified. For example, a direct proportionality between stiffness and connective tissue percentage was ascertained. Micropipette aspiration technique has been applied to measure selectively the mechanical properties of the top layer of an intact multilayer biomaterial [22].

Mathematical modeling of biological tissues has been reviewed [23] focusing particularly on the stress-strain responses of biological tissues subjected to mechanical loading. Continuum Mechanics has been also applied to interpret deformation profile of aspirated soft tissues and so to evaluate their mechanical properties [24,25]. Micropipette aspiration has been studied by using finite element analysis simulation of the sample aspiration into the micropipette [26] and by considering also the influence of the sample adhesion. An inverse finite-element method strategy has been used in 2007 [27] to evaluate the elastic modulus and yield properties of human cortical specimens of the radial diaphysis and some numerical values have been proposed and assessed by means of isotropic linear-elastic simulations. The mechanical response of soft biological materials to nanoindentation with spherical indenters has been investigated by using finite element simulations together with three different material constitutive laws, namely, elastic, isotropic hyperelastic and anisotropic hyperelastic [28], in order to improve measurements offered by nanoindentation experiments. A computational tool has been developed [29] to describe and predict the mechanical behavior of electrospun scaffolds characterized by different microstructures. This has been useful to study the global mechanical properties of cardiovascular valve-shaped scaffolds and to understand that anisotropy is necessary to reproduce the deformation patterns observed in native heart valves.

Considering that cells are the fundamental building blocks of tissue, a more detailed comprehension of tissue mechanics can be achieved by studying the Mechanics of cells. Any modification of a cell mechanical characteristic is related to a change in its functionality, possible pathological diseases, or response to the environment. A review on cell mechanics [30] pointed out several relations between cell functionality and its mechanical response and presented theoretical models together with experimental and computational approaches. For example, Ekpenyong et al. [31] showed that some cells alter their viscoelastic properties during stem cell differentiation. The modification has been measured using an optical stretcher. Gossett et al. [32] illustrated an automated microfluidic technology capable of probing single–cell deformability at approximately 2000 cells/s. A microfluidic chip has been also proposed [33] to apply mechanical stress to live cells and measure their nuclear deformability, with the purpose of characterizing the nuclear response to physical stress. Nucleus physical plasticity has been also studied in stem cell differentiation [34]. The mechanical behavior has been studied also to characterize Embryonic stem cells (ESCs) [35]. Furthermore, optical deformability has been used to monitor the subtle changes during the progression of mouse fibroblasts and human breast epithelial cells from normal to cancerous state [36]. Finally, real-time deformability cytometry (RT-DC) has been introduced [37] for continuous cell mechanical characterization of large populations (>100,000 cells) with analysis rates greater than 100 cells/s.

Cell-specific mechanical properties have a great influence on cell stretching response. By using this concept, a method to distinguish cell types has been developed, based on stretching tests performed by positioning cells between microelectrodes and applying dielectrophoretic forces [38].

Cellular response to mechanical stimuli has been investigated in [39] where an electromagnetic cell stretching platform, based on double-sided stretching axes, has been used to introduce a cyclic and static strain pattern on a cell culture.

In the present investigation the mechanical handling of a tissue sample has been suggested as a method to test the tissue stiffness and viscous damping response. According to the suggested procedure, the handling operation is carried out by means of a microgripper that has been independently fabricated for this dedicated purpose and makes use of a new concept so–called *CSFH* hinge [40,41,42].

A new mechanical model for determining the tissue stiffness and viscosity has been developed, based on Kinematics and Continuum Mechanics. Furthermore, a control strategy has been proposed and simulated. As shown in literature [43,44,45], grippers and microgrippers have been extensively developed during the last decades and, more recently, new synthesis techniques [46,47,48,49] allowed designers to handle tissue also by selective, non isotropic compliance [50]. A former version of microgripper for tissue manipulation has been presented in 2015 [51], while this paper presents a new refined version of the hardware and a new control system for measuring both the stiffness and the viscosity coefficient of the tissue sample.

## 2. The Adopted Microsystem

**Figure 1:**
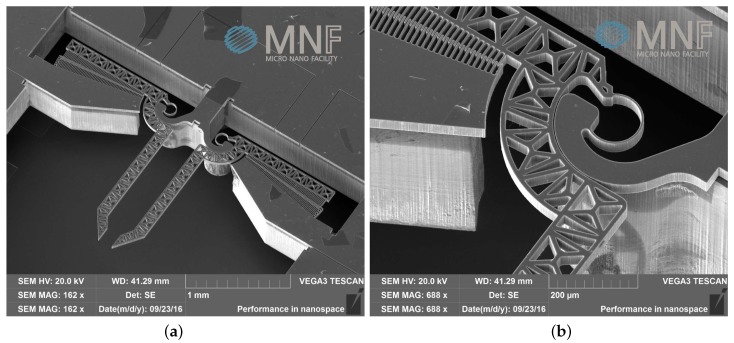
A SEM image of (**a**) the mechanical component of the microsystem and (**b**) a detailed vew of the CSFH hinge.

A new microsystem has been specifically designed and fabricated for the purpose of this investigation. The microsystem has been also provided with a dedicated control algorithm for testing the mechanical characteristics of any biological tissue sample at the micro or nanoscale. Therefore, the control method is intended as applied to the microsystem depicted in Figure 1a,b. The actual fabrication method made use of Deep Reactive-Ion Etching applied on Silicon on Insulator wafer. The fabrication process is described in detail in [52].

Mobility is provided to the microsystem by two CSFHs, Conjugate Surface Flexure Hinges, that will be briefly introduced in the next section.

## 3. The Modeling Approach

As mentioned in the Introduction, many investigations presented in the literature were focused on the development of models to understand cell mechanics, such as cortical shell–liquid core models and solid models [53]. Generally, the mechanical model depends on the experimental approach, that can be based on force application techniques or force sensing techniques [30]. Also in this investigation, the mechanical model of the tissue depends on experimental techniques, as described hereafter.

**Figure 2:**
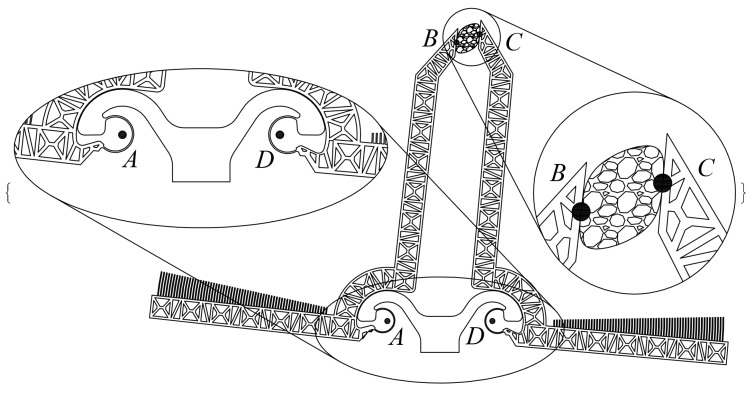
The gripping system in a generic configuration.

A fundamental component of the developed microsystem can be represented as in Figure 2, where the mobile jaws show up in a possible configuration. A controlled-desired displacement of the right hand side jaw can be obtained by actuating the left hand side jaw, through the elastic response of a grabbed tissue sample. By virtue of the adopted CSFH Conjugate Surface Flexure Hinge it could be shown that points A and D are quite stationary during actuation and therefore they can be considered the rotation centers of the two jaws [54,55]. Considering also that the two jaws are rather stiffer than the tissue samples, the four-bar linkage ABCD can be adopted to model the whole microsystem, provided that the coupler link BC is allowed to have a variable length.

**Figure 3:**
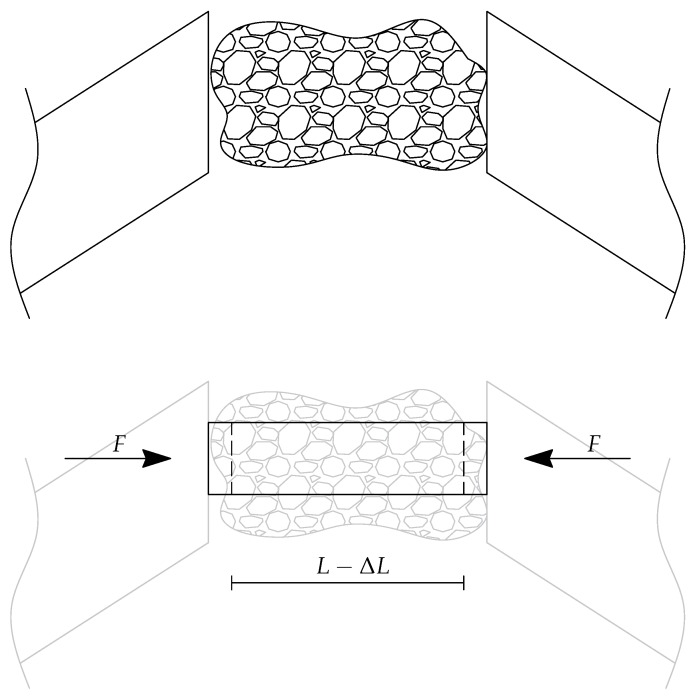
Modeling of tissue sample.

More specifically, the tissue sample is modeled by using the continuum mechanics theory, as a homogeneous and isotropic material. According to the multi-scale, three-level hierarchical approach for mechanotransduction proposed by Lim et al. [53], the model proposed in this Section can be classified at Level 1. Therefore, the tissue sample is considered as a beam element with constant cross-section (with area A) subjected to the axial load F. With reference to Figure 3, the normal strain is defined as ϵ=ΔL/L, whereas the normal stress is equal to σ=F/A. Assuming a linear elastic behaviour for the material, according to the Hooke’s law the stress-strain relationship can be written as σ=Eϵ, where E is the Young modulus. By using the definitions of normal stress and normal strain, the previous equation can be written as

(1) $$\frac{F}{A}=E\frac{\Delta L}{L},$$

and the stiffness coefficient of the tissue sample can be obtained as

(2) $$k=\frac{EA}{L}.$$

**Figure 4:**
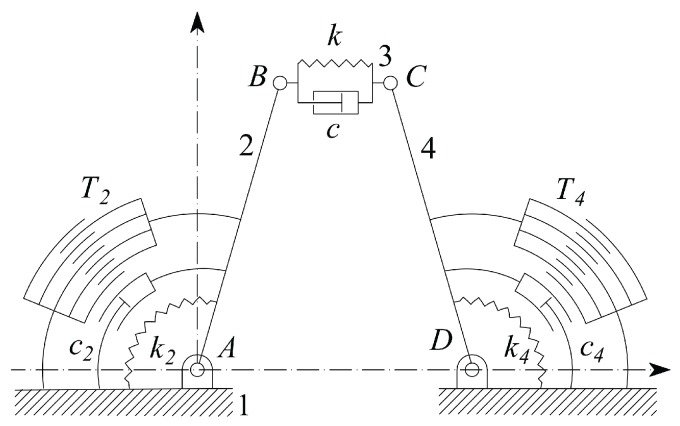
The schematic gripper layout in the initial configuration.

It is worth noticing also that the sample coefficient of viscosity c is not coincident to the material viscosity μ, because c depends on the geometry of the sample. In general c is linearly proportional to μ, as, for example, for the simple cylindrical viscous damper [56]. Therefore, the actual value of c must be related to the tissue material viscosity through a model, which involves also the sample geometry. In this contribution the sample has been modeled as a linear spring working in parallel with a viscous damper. The actual system configuration will be described by means of initial, target and incremental variables according to the following Nomenclature, which makes reference to Figure 4.

**Figure 5:**
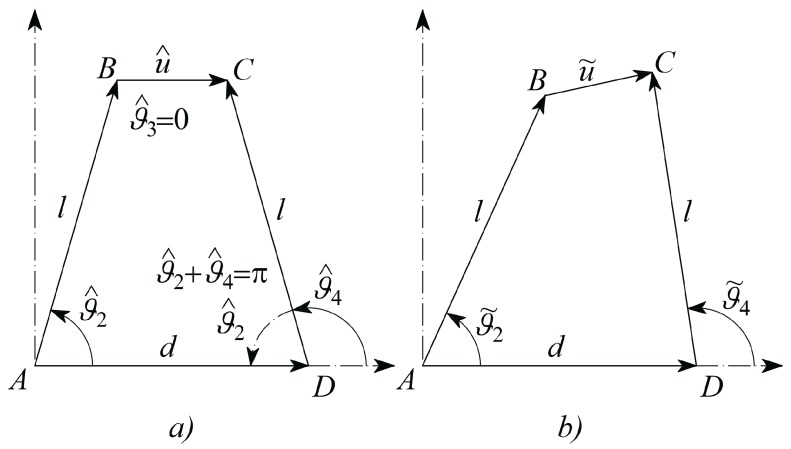
The micro–system closed loop chain in the initial (**a**) and in the generic (**b**) configuration.

- hat ^ refers to a constant parameter, such as, for example, those referring to the initial configuration, as shown in Figure 5a;
- tilde^˜^ refers to an actual parameter at the generic configuration, as represented in Figure 5b;
- superscript${}^{0}$ refers to the *desired* or *target* parameter;
- angles of bars are measured counterclockwise, starting from the positive abscissa;
- $\tilde{u}=\hat{u}+u$ is the length of vector $\vec{BC}$ which is split in the initial length $\hat{u}$ and the deformation u;
- $\vartheta_{2}$, $\vartheta_{3}$ and $\vartheta_{4}$ refer to the variations of the angular positions of the link vectors $\vec{AB}$, $\vec{BC}$ and $\vec{DC}$, with respect to their initial position; in this way their actual absolute angular positions will be $\tilde{\vartheta_{2}}={\hat{\vartheta }}_{2}+\vartheta_{2}$, ${\tilde{\vartheta }}_{3}={\hat{\vartheta }}_{3}+\vartheta_{3}$, ${\tilde{\vartheta }}_{4}={\hat{\vartheta }}_{4}+\vartheta_{4}$, respectively;
- ${\hat{\vartheta }}_{2}=\pi -{\hat{\vartheta }}_{4}$ (as Figure 5a shows that they are *supplementary* angles)
- $\hat{u}=d-2l\cos{\hat{\vartheta }}_{2}$, from geometry represented in Figure 5a;
- l is the common length of the two vectors $\vec{AB}$ and $\vec{DC}$;
- d is the length of the frame link AD;
- k is the stiffness coefficient of the tissue sample;
- $k_{2}$ and $k_{4}$ are the two jaws torsional stiffness, which are related to the CSFH curved beam material and geometry;
- $r_{b}$, b, h and β are the radius, width, thickness and beam subtended angle of the CSFH flexure curved beam;
- c, $c_{2}$ and $c_{4}$ represent the viscous damping coefficients of the sample and of the two jaws;
- $I_{2}$ and $I_{4}$ represents the two jaws moments of inertia around A and D, with $I_{2}=I_{4}$;
- $v_{2}$ and $v_{4}$ are the tensions applied to the comb drives;
- χ, g and w are the overlap angle, gap and width of the comb drive fingers;
- $z_{0}$ device-handle gap (silicon oxide layer thickness);
- μ air viscosity at 25 °C;
- $J_{p\;2,4}$ polar moment of area exposed to air viscous damping, calculated around the rotation points;
- ${\hat{R}}_{a}$ equivalent radius employed to model the air viscous damping.

Parameter values given in input to the developed model are shown in Table 1.

Air viscous damping has been used to model friction on the mobile jaws. Slide-film damping with continuum flow regime has been adjusted for the case of rotary and alternate motion.

In case of rotary motion and assuming $z_{0}$ one order of magnitude smaller than the comb drive maximum (external) radius $r_{\max}$, the viscous tangential traction $dT=\mu \cdot dA\cdot \frac{r\dot{\vartheta }}{z_{0}}$ on the elementary surface dA at the distance r from the rotation axis, exerts an elementary moment $dm=r\cdot dT=r\cdot \mu dA\cdot \frac{r\dot{\vartheta }}{z_{0}}$. Therefore overall moment is approximately equal to $\frac{\mu }{z_{0}}\cdot \dot{\vartheta }\cdot \int_{r_{\min}}^{r_{\max}}r^{2}dA=c\cdot \dot{\vartheta }$. For this reason, the viscous damping coefficients $c_{2}$ and $c_{4}$ can be estimated as

(3) $$c_{2,4}=\frac{\mu_{e}J_{p\;2,4}}{z_{0}}$$

for either jaw 2 or 4, where the polar moments of area $J_{p\;2}$ and $J_{p\;4}$ have been calculated around the rotation points and by taking into account the exact geometry of the rotating masses. The *effective* viscosity $\mu_{e}$ has been calculated as

(4) $$\mu_{e}=\frac{\mu }{1+2K_{n}},$$

where $K_{n}$ is the Knudsen number, equal to the ratio of the mean free path of the molecules in air, λ, to the critical dimension of the device, $d_{c}$. In our case the length λ is equal to 0.07μm because the system works at $10^{5}$ Pa, whereas $d_{c}$ is equal to 2μm, namely, the gap between the conjugate surfaces. Since $K_{n}≃3.5\times 10^{-2}$, the continuum flow model can be accepted as the actual regime with $\mu_{e}≃\mu$.

## 4. The Adopted Electromechanical Model

Since the coupler BC has variable length, in this particular case the four-bar linkage has two DoF (Degrees of Freedom). Considering that two rotary comb drives give us control over $\vartheta_{2}$ and $\vartheta_{4}$, these two variables can be assumed to be the independent ones. However, for the sake of the present investigation, the interest is in u and $\dot{u}$. The former can be calculated by extracting the real and imaginary components of vector $\vec{BC}$ and then evaluating its module $\left|\vec{BC}\right|=\tilde{u}$ and inclination angle $\vartheta_{3}$.

In fact, given ${\tilde{\vartheta }}_{2}$, ${\tilde{\vartheta }}_{3}=\vartheta_{3}$, ${\tilde{\vartheta }}_{4}$ and $\tilde{u}$, the closed loop complex equation

(5) $$le^{i{\tilde{\vartheta }}_{2}}+\tilde{u}e^{i\vartheta_{3}}-le^{i\tilde{\vartheta_{4}}}-d=0$$

allows $\vec{BC}\equiv \tilde{u}e^{i\vartheta_{3}}$ to be expressed as

(6) $$d-le^{i{\tilde{\vartheta }}_{2}}+le^{i{\tilde{\vartheta }}_{4}},$$

and therefore to obtain immediately its real

(7) $$ℜ\left(\vec{BC}\right)=d-l\cos{\tilde{\vartheta }}_{2}+l\cos{\tilde{\vartheta }}_{4}$$

and imaginary

(8) $$ℑ\left(\vec{BC}\right)=-l\sin{\tilde{\vartheta }}_{2}+l\sin{\tilde{\vartheta }}_{4}$$

components. From these, the values of $\tilde{u}$ and $\vartheta_{3}$ can be computed, for all configuration during contact, as functions of ${\tilde{\vartheta }}_{2}$ and ${\tilde{\vartheta }}_{4}$ according to the expressions

(9) $$\tilde{u}=\sqrt{{ℜ}^{2}\left(\vec{BC}\right)+{ℑ}^{2}\left(\vec{BC}\right)}$$

and

(10) $$\vartheta_{3}=\arctan\frac{ℑ\left(\vec{BC}\right)}{ℜ\left(\vec{BC}\right)}$$

Reminding that $\tilde{u}=\hat{u}+u$ and multiplying the first time derivative of Equation (5)

(11) $$i{\dot{\vartheta }}_{2}le^{i{\tilde{\vartheta }}_{2}}+i\tilde{u}{\dot{\vartheta }}_{3}e^{i\vartheta_{3}}+\dot{u}e^{i\vartheta_{3}}-i{\dot{\vartheta }}_{4}le^{i{\tilde{\vartheta }}_{4}}=0$$

by $e^{-i\vartheta_{3}}$, the complex equation

(12) $$i{\dot{\vartheta }}_{2}le^{i\left({\tilde{\vartheta }}_{2}-\vartheta_{3}\right)}+\tilde{u}i{\dot{\vartheta }}_{3}+\dot{u}-i{\dot{\vartheta }}_{4}le^{i\left({\tilde{\vartheta }}_{4}-\vartheta_{3}\right)}=0$$

is obtained, where the real components can be arranged to express the deformation velocity of the sample tissue

(13) $$\dot{u}={\dot{\vartheta }}_{2}l\sin\left({\tilde{\vartheta }}_{2}-\vartheta_{3}\right)-{\dot{\vartheta }}_{4}l\sin\left({\tilde{\vartheta }}_{4}-\vartheta_{3}\right)$$

which does not depend directly on ${\dot{\vartheta }}_{3}$.

Assuming that a jaw and its corresponding comb teeth mobile set can be approximated as a whole *pseudo–rigid* system, Euler’s rotation equation can be written twice, to express the dynamic balance of the left and right jaw systems during rotations, namely,

(14) $$\begin{array}{cc} T_{2}\left(v_{2}\right) & =I_{2}{\ddot{\vartheta }}_{2}+cl\sin\left({\tilde{\vartheta }}_{2}-\vartheta_{3}\right)\dot{u}+c_{2}{\dot{\vartheta }}_{2}+kl\sin\left({\tilde{\vartheta }}_{2}-\vartheta_{3}\right)u+k_{2}\vartheta_{2}, \end{array}$$

(15) $$\begin{array}{cc} T_{4}\left(v_{4}\right) & =I_{4}{\ddot{\vartheta }}_{4}-cl\sin\left({\tilde{\vartheta }}_{4}-\vartheta_{3}\right)\dot{u}+c_{4}{\dot{\vartheta }}_{4}-kl\sin\left({\tilde{\vartheta }}_{4}-\vartheta_{3}\right)u+k_{4}\vartheta_{4}, \end{array}$$

where the sample inertia has been assumed to be negligible.

**Figure 6:**
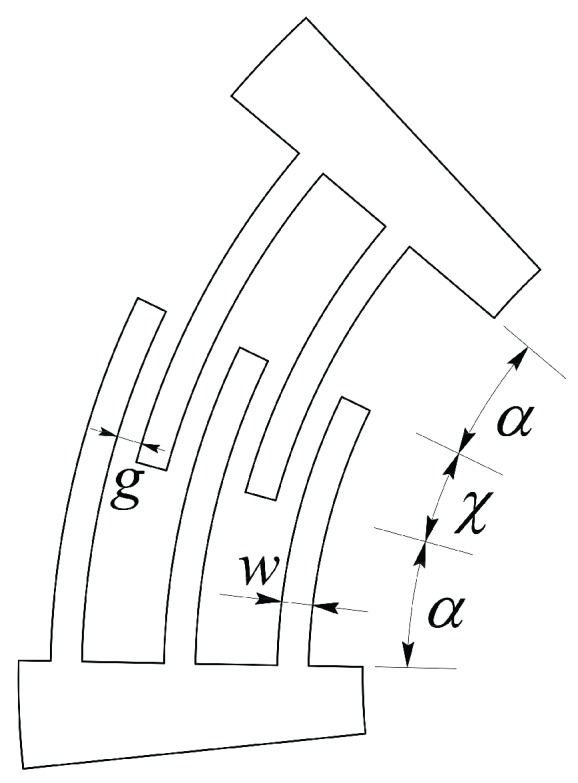
Parameters used for the configuration of the comb drives.

With reference to Figure 6, by introducing the comb drives equivalent radius

(16) $$\hat{R}=\rho r_{\max}=\frac{g}{2n}\;\left[\sum_{k=1}^{n-1}{\left[\ln A_{k}\right]}^{-1}+\sum_{k=0}^{n-1}{\left[\ln B_{k}\right]}^{-1}\right]$$

where $r_{\max}=r_{0}+2n\left(g+w\right)$ and (see also ref. [57,58])

$$\begin{array}{cc} A_{k} & =\frac{r_{0}+2k\left(w+g\right)}{r_{0}+2k\left(w+g\right)-g},\\ B_{k} & =\frac{r_{0}+\left(2k+1\right)\left(w+g\right)}{r_{0}+2k\left(w+g\right)+w}. \end{array}$$

the torque and the capacitance at the two comb drives 2 and 4 can be expressed, respectively, as

$$\left\{\begin{array}{l} T_{j}=\hat{R}n\frac{\epsilon_{0}hv_{j}^{2}}{g}\\ C_{j}\left(\vartheta_{j}\right)=\frac{2\varepsilon h\hat{R}\left(\chi \mp \vartheta_{j}\right)}{g},\\ \mathrm{with}\;j=2,4, \end{array}\right.$$

where minus or plus holds for j=2 or j=4, respectively.

## 5. The Identification of the Sample Stiffness and the Damping Coefficients

The technique proposed for measuring the damping coefficient of a sample tissue consists in exciting it with a variable compressive force which is characterized by a suitable wave form. Then, after a first phase during which the tissue is grabbed and kept firmly between the jaws, a torque is applied and the system response is measured.

### 5.1. Characterization of the Sample Stiffness

More precisely, if a constant torque $T_{2}\left(v_{2}\right)=\tau_{2}^{0}\neq 0$ is applied, while $T_{4}\left(v_{4}\right)=0$, with $\tau_{2}^{0}$ large enough to grab a sample between the jaws, keeping it firmly but without damaging it, a small variation of ${\tilde{\vartheta }}_{4}$ is obtained; the amplitude of such variation is directly related to the gripping force applied on the sample.

**Figure 7:**
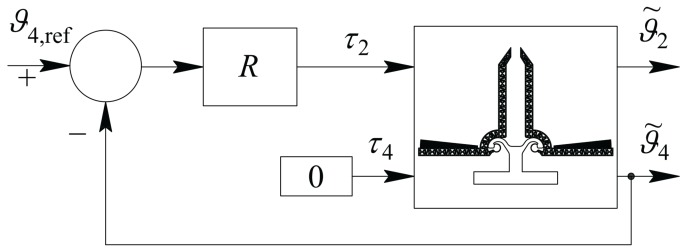
Control scheme adopted for evaluating stiffness k.

This effect can be obtained with a manual operation, in which the input voltage $v_{2}$ is slowly increased, in a quasi-static condition, until the desired angle ${\tilde{\vartheta }}_{4}^{0}$ is reached, or under a control system, which brings the ${\tilde{\vartheta }}_{4}$ angle to the desired value ${\tilde{\vartheta }}_{4,ref}={\tilde{\vartheta }}_{4}^{0}$. In this paper, we will design the control scheme accordingly to the second option, as represented in Figure 7, where block R represents a Regulator. In the case under study, R consists of a PID (Proportional-Integral-Derivative standard regulator), and represents the control block which generates the input torque $\tau_{2}$ on the basis of the position error, between the desired angle $\theta_{4,ref}$ and the actual one ${\tilde{\theta }}_{4}$, until it goes, asymptotically, to zero.

**Figure 8:**
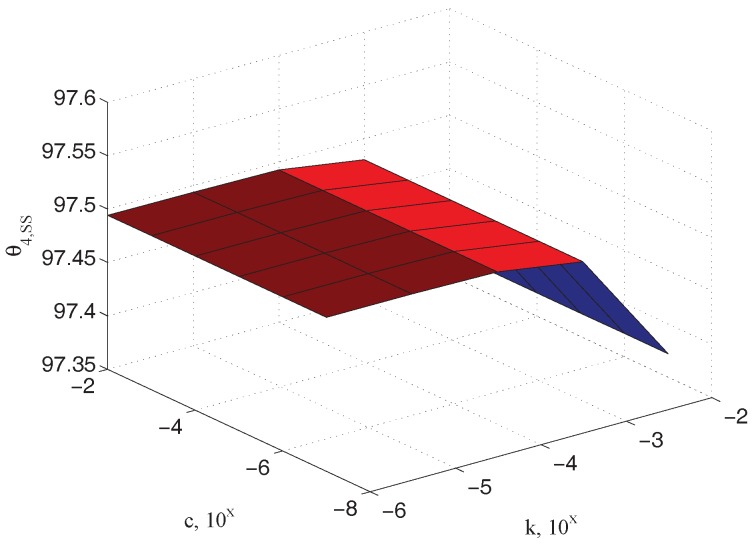
Dependency of the steady state angle $\theta_{4,ss}$ on stiffness k (note that viscosity c does not affect steady state balance).

**Figure 9:**
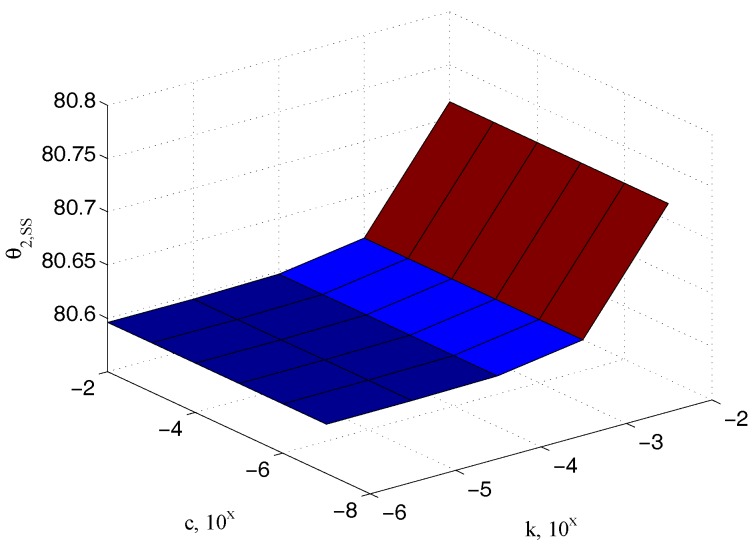
Dependency of the steady state angle $\theta_{2,ss}$ on k.

**Figure 10:**
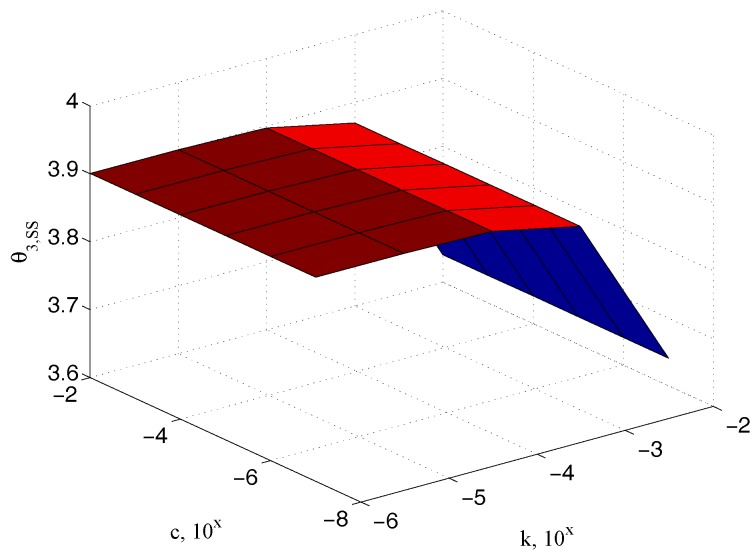
Dependency of the steady state angle $\theta_{3,ss}$ on k.

Figure 8 depicts the steady state values of the output measured angle $\theta_{4,ss}$ as a function of the unknown parameters k and c (In the following simulations the parameter values indicated in Table 1 have been used. The plane dimensions of the specimen are approximately $50\times 10^{-6}$ m × $200\times 10^{-6}$ m), confirming the physical observation that it has to be dependent on the elastic coefficient only. Similar relationships can be referred to the input $\theta_{2,ss}$ and the sample $\theta_{3,ss}$ angles, as reported in Figure 9 and Figure 10, respectively.

In any case, at steady state, the Equations (14) and (15) assume the expressions

(17) $$\begin{array}{ccc} \tau_{2}^{0} & = & kl\sin\left({\tilde{\vartheta }}_{2}^{0}-\vartheta_{3}^{0}\right)u^{0}+k_{2}\vartheta_{2}^{0} \end{array}$$

(18) $$\begin{array}{ccc} 0 & = & -kl\sin\left({\tilde{\vartheta }}_{4}^{0}-\vartheta_{3}^{0}\right)u^{0}+k_{4}\vartheta_{4}^{0} \end{array}$$

where ${\tilde{\vartheta }}_{i}^{0}={\hat{\vartheta }}_{i}+\vartheta_{i}^{0}$, i=2,3,4 and $u^{0}=\tilde{u}-\hat{u}$. Note that, in these expressions, both $u^{0}$ and $\vartheta_{3}^{0}$ can be computed from (9) and (10), once the values of ${\tilde{\vartheta }}_{2}^{0}$ and ${\tilde{\vartheta }}_{4}^{0}$ are measured.

### 5.2. Characterization of Sample Characteristic Damping

Let us choose, now, in (14), $T_{2}\left(v_{2}\right)=\tau_{2}^{0}+\tau_{2}$, with $\tau_{2}:ℜ\Rightarrow ℜ$ a suitable time function described in the following; in this case, Equation (14), using Equation (17), becomes

(19) $$\begin{array}{cc} \tau_{2} & =I_{2}{\ddot{\vartheta }}_{2}+cl\sin\left({\tilde{\vartheta }}_{2}-\vartheta_{3}\right)\dot{u}+c_{2}{\dot{\vartheta }}_{2}+kl\sin\left({\tilde{\vartheta }}_{2}-\vartheta_{3}\right)u-kl\sin\left({\tilde{\vartheta }}_{2}^{0}-\vartheta_{3}^{0}\right)u_{0}+k_{2}\vartheta_{2}-k_{2}\vartheta_{2}^{0} \end{array}$$

Some computations can be performed to put in evidence the dependency of the steady state output under a particular choice of input τ. Initially, the case of small and slow signals is addressed, bringing to a linearly approximated analysis useful to show, at a first approximation, the relationships between the values of the viscoelastic parameters and the measured output signal; the generalization of the results, involving both the nonlinear terms of the models and possible nonlinearities of the parameters, is then illustrated through some simulations results, obtained considering the fully nonlinear system.

Then, setting

(20) $$\begin{array}{ccc} {\tilde{\vartheta }}_{2}= & {\tilde{\vartheta }}_{2}^{0}+\delta \vartheta_{2},\;\vartheta_{3}= & \vartheta_{3}^{0}+\delta \vartheta_{3}\\ {\tilde{\vartheta }}_{4}= & {\tilde{\vartheta }}_{4}^{0}+\delta \vartheta_{4},\;u= & u^{0}+\delta u \end{array}$$

and, as a consequence,

(21) $${\dot{\vartheta }}_{i}=\dot{\delta \vartheta_{i}},\;{\ddot{\vartheta }}_{i}=\ddot{\delta \vartheta_{i}},\;i=2,3,4,\;\dot{u}=\dot{\delta u}$$

it is possible to rewrite Equation (19) as:

(22) $$\begin{array}{c} I_{2}\ddot{\delta \vartheta_{2}}+cl\sin\left({\tilde{\vartheta }}_{2}^{0}+\delta \vartheta_{2}-\vartheta_{3}^{0}-\delta \vartheta_{3}\right)\dot{\delta u}+kl\sin\left({\tilde{\vartheta }}_{2}^{0}+\delta \vartheta_{2}-\vartheta_{3}^{0}-\delta \vartheta_{3}\right)\left(u^{0}+\delta u\right)+\\ -kl\sin\left({\tilde{\vartheta }}_{2}^{0}-\vartheta_{3}^{0}\right)u^{0}+c_{2}\dot{\delta \vartheta_{2}}+k_{2}\delta \vartheta_{2}=\tau_{2} \end{array}$$

Under the hypothesis of $∥\tau_{2}∥\ll \tau_{2}^{0}$, ∀t, small enough to have $\delta \vartheta_{i}\ll {\tilde{\vartheta }}_{i}^{0}$, i=2,3,4, and $\delta u\ll u_{0}$, the Taylor series approximation, up to the first order, can be introduced

(23) $$\sin\left({\tilde{\vartheta }}_{2}^{0}+\delta \vartheta_{2}-\vartheta_{3}^{0}-\delta \vartheta_{3}\right)≃\sin\left({\tilde{\vartheta }}_{2}^{0}-\vartheta_{3}^{0}\right)+\cos\left({\tilde{\vartheta }}_{2}^{0}-\vartheta_{3}^{0}\right)\left(\delta \vartheta_{2}-\delta \vartheta_{3}\right)$$

which, once used in Equation (22), gives the approximated expression

(24) $$\begin{array}{c} I_{2}\ddot{\delta \vartheta_{2}}+cl\left[\sin\left({\tilde{\vartheta }}_{2}^{0}-\vartheta_{3}^{0}\right)+\cos\left({\tilde{\vartheta }}_{2}^{0}-\vartheta_{3}^{0}\right)\left(\delta \vartheta_{2}-\delta \vartheta_{3}\right)\right]\dot{\delta u}+\\ kl\left[\sin\left({\tilde{\vartheta }}_{2}^{0}-\vartheta_{3}^{0}\right)+\cos\left({\tilde{\vartheta }}_{2}^{0}-\vartheta_{3}^{0}\right)\left(\delta \vartheta_{2}-\delta \vartheta_{3}\right)\right]\left(u^{0}+\delta u\right)+\\ -kl\sin\left({\tilde{\vartheta }}_{2}^{0}-\vartheta_{3}^{0}\right)u^{0}+c_{2}\dot{\delta \vartheta_{2}}+k_{2}\delta \vartheta_{2}=\tau_{2} \end{array}$$

Performing all the computations, the latter expression can be expanded as

(25) $$\begin{array}{c} I_{2}\ddot{\delta \vartheta_{2}}+cl\sin\left({\tilde{\vartheta }}_{2}^{0}-\vartheta_{3}^{0}\right)\dot{\delta u}+kl\sin\left({\tilde{\vartheta }}_{2}^{0}-\vartheta_{3}^{0}\right)\left(u^{0}+\delta u\right)-kl\sin\left({\tilde{\vartheta }}_{2}^{0}-\vartheta_{3}^{0}\right)u^{0}+\\ +kl\cos\left({\tilde{\vartheta }}_{2}^{0}-\vartheta_{3}^{0}\right)\left(\delta \vartheta_{2}-\delta \vartheta_{3}\right)u^{0}+cl\cos\left({\tilde{\vartheta }}_{2}^{0}-\vartheta_{3}^{0}\right)\left(\delta \vartheta_{2}-\delta \vartheta_{3}\right)\dot{\delta u}+\\ +kl\cos\left({\tilde{\vartheta }}_{2}^{0}-\vartheta_{3}^{0}\right)\left(\delta \vartheta_{2}-\delta \vartheta_{3}\right)\delta u+c_{2}\dot{\delta \vartheta_{2}}+k_{2}\delta \vartheta_{2}=\tau_{2} \end{array}$$

After simplifications, Equation (25) can be reordered as

(26) $$\begin{array}{c} I_{2}\ddot{\delta \vartheta_{2}}+cl\sin\left({\tilde{\vartheta }}_{2}^{0}-\vartheta_{3}^{0}\right)\dot{\delta u}+kl\sin\left({\tilde{\vartheta }}_{2}^{0}-\vartheta_{3}^{0}\right)\delta u+\\ +kl\cos\left({\tilde{\vartheta }}_{2}^{0}-\vartheta_{3}^{0}\right)\left(\delta \vartheta_{2}-\delta \vartheta_{3}\right)u^{0}+c_{2}\dot{\delta \vartheta_{2}}+k_{2}\delta \vartheta_{2}+\\ +cl\cos\left({\tilde{\vartheta }}_{2}^{0}-\vartheta_{3}^{0}\right)\left(\delta \vartheta_{2}-\delta \vartheta_{3}\right)\dot{\delta u}+kl\cos\left({\tilde{\vartheta }}_{2}^{0}-\vartheta_{3}^{0}\right)\left(\delta \vartheta_{2}-\delta \vartheta_{3}\right)\delta u=\tau_{2} \end{array}$$

where the terms corresponding to an approximation of order one are collected and separated from the others. Neglecting once more, as done in Equation (23), all the terms of approximated order greater than one, the linear approximation of Equation (19) assumes the final expression

(27) $$\begin{array}{c} I_{2}\ddot{\delta \vartheta_{2}}+cl\sin\left({\tilde{\vartheta }}_{2}^{0}-\vartheta_{3}^{0}\right)\dot{\delta u}+kl\sin\left({\tilde{\vartheta }}_{2}^{0}-\vartheta_{3}^{0}\right)\delta u+kl\cos\left({\tilde{\vartheta }}_{2}^{0}-\vartheta_{3}^{0}\right)\left(\delta \vartheta_{2}-\delta \vartheta_{3}\right)u^{0}+\\ +c_{2}\dot{\delta \vartheta_{2}}+k_{2}\delta \vartheta_{2}=\tau_{2}. \end{array}$$

The same considerations can be made for Equation (15), under zero input and once Equation (18) is considered. After easy computations one gets for the linearized approximated expression up to the first order

(28) $$\begin{array}{c} I_{4}\ddot{\delta \vartheta_{4}}-cl\sin\left({\tilde{\vartheta }}_{4}^{0}-\vartheta_{3}^{0}\right)\dot{\delta u}-kl\sin\left({\tilde{\vartheta }}_{4}^{0}-\vartheta_{3}^{0}\right)\delta u-kl\cos\left({\tilde{\vartheta }}_{4}^{0}-\vartheta_{3}^{0}\right)\left(\delta \vartheta_{4}-\delta \vartheta_{3}\right)u^{0}+\\ +c_{4}\dot{\delta \vartheta_{4}}+k_{4}\delta \vartheta_{4}=0 \end{array}$$

The Laplace transforms L of Equations (27) and (28) can be performed; recalling that given a Laplace transformable time function f(t), with f(0)=0 and $\dot{f}\left(0\right)=0$, one has that $L\left\{f(t)\right\}=F\left(s\right)$, $L\left\{\dot{f}\left(t\right)\right\}=sF\left(s\right)$ and $L\left\{\ddot{f}\left(t\right)\right\}=s^{2}F\left(s\right)$, Equation (27) becomes:

(29) $$\begin{array}{c} I_{2}s^{2}\delta \vartheta_{2}\left(s\right)+\left(cls+kl\right)\sin\left({\tilde{\vartheta }}_{2}^{0}-\vartheta_{3}^{0}\right)\delta u\left(s\right)+kl\cos\left({\tilde{\vartheta }}_{2}^{0}-\vartheta_{3}^{0}\right)u^{0}\left(\delta \vartheta_{2}\left(s\right)-\delta \vartheta_{3}\left(s\right)\right)+\\ +c_{2}s\delta \vartheta_{2}\left(s\right)+k_{2}\delta \vartheta_{2}\left(s\right)=\tau_{2}\left(s\right) \end{array}$$

while Equation (28) is transformed into

(30) $$\begin{array}{c} I_{4}s^{2}\delta \vartheta_{4}\left(s\right)-\left(cls+kl\right)\sin\left({\tilde{\vartheta }}_{4}^{0}-\vartheta_{3}^{0}\right)\delta u\left(s\right)-kl\cos\left({\tilde{\vartheta }}_{4}^{0}-\vartheta_{3}^{0}\right)u^{0}\left(\delta \vartheta_{4}\left(s\right)-\delta \vartheta_{3}\left(s\right)\right)+\\ +c_{4}s\delta \vartheta_{4}\left(s\right)+k_{4}\delta \vartheta_{4}\left(s\right)=0 \end{array}$$

According to Equations (9) and (10), $\delta \vartheta_{3}\left(s\right)$ and δu(s) can be explicitly written as functions of $\delta \vartheta_{2}\left(s\right)$ and $\delta \vartheta_{4}\left(s\right)$ computing the first order series expansion of (9) and (10):

(31) $$\tilde{u}=u^{o}+\delta u≃u^{0}+{\left.\frac{\partial \tilde{u}}{\partial {\tilde{\vartheta }}_{2}}\right|}^{0}\delta \vartheta_{2}+{\left.\frac{\partial \tilde{u}}{\partial {\tilde{\vartheta }}_{4}}\right|}^{0}\delta \vartheta_{4}$$

and

(32) $$\vartheta_{3}=\vartheta_{3}^{0}+\delta \vartheta_{3}≃\vartheta_{3}^{0}+{\left.\frac{\partial \vartheta_{3}}{\partial {\tilde{\vartheta }}_{2}}\right|}^{0}\delta \vartheta_{2}+{\left.\frac{\partial \vartheta_{3}}{\partial {\tilde{\vartheta }}_{4}}\right|}^{0}\delta \vartheta_{4}$$

where ${\left.f({\tilde{\vartheta }}_{2},{\tilde{\vartheta }}_{4})\right|}^{0}$ denotes the evaluation of function $f({\tilde{\vartheta }}_{2},{\tilde{\vartheta }}_{4})$ at ${\tilde{\vartheta }}_{i}=\vartheta_{i}^{0}$, i=2,4, i.e., $f({\tilde{\vartheta }}_{2}^{0},{\tilde{\vartheta }}_{4}^{0})$. Explicitly, recalling expressions (7) and (8), one gets

(33) $$\begin{array}{cc} {\left.\frac{\partial \tilde{u}}{\partial {\tilde{\vartheta }}_{2}}\right|}^{0} & ={\left.\frac{2ℜ\left(\right)\frac{\partial ℜ()}{\partial {\tilde{\vartheta }}_{2}}+2ℑ\left(\right)\frac{\partial ℑ()}{\partial {\tilde{\vartheta }}_{2}}}{2\sqrt{{ℜ}^{2}\left(\right)+{ℑ}^{2}\left(\right)}}\right|}^{0}=\\ & ={\left.\frac{\left(d-l\cos{\tilde{\vartheta }}_{2}+l\cos{\tilde{\vartheta }}_{4}\right)l\sin{\tilde{\vartheta }}_{2}+\left(-l\sin{\tilde{\vartheta }}_{2}+l\sin{\tilde{\vartheta }}_{4}\right)\left(-l\cos{\tilde{\vartheta }}_{2}\right)}{\sqrt{{ℜ}^{2}\left(\cdot \right)+{ℑ}^{2}\left(\cdot \right)}}\right|}^{0}=\\ & ={\left.\frac{dl\sin{\tilde{\vartheta }}_{2}+l^{2}\left(+\sin{\tilde{\vartheta }}_{2}\cos{\tilde{\vartheta }}_{4}-\sin{\tilde{\vartheta }}_{4}\cos{\tilde{\vartheta }}_{2}\right)}{\sqrt{{ℜ}^{2}\left(\cdot \right)+{ℑ}^{2}\left(\cdot \right)}}\right|}^{0}={\left.\frac{dl\sin{\tilde{\vartheta }}_{2}+l^{2}\sin\left({\tilde{\vartheta }}_{2}-{\tilde{\vartheta }}_{4}\right)}{\sqrt{{ℜ}^{2}\left(\cdot \right)+{ℑ}^{2}\left(\cdot \right)}}\right|}^{0} \end{array}$$

Analogous computations bring to the expressions

(34) $${\left.\frac{\partial \tilde{u}}{\partial {\tilde{\vartheta }}_{4}}\right|}^{0}={\left.\frac{-dl\sin{\tilde{\vartheta }}_{4}-l^{2}\sin\left({\tilde{\vartheta }}_{2}-{\tilde{\vartheta }}_{4}\right)}{\sqrt{{ℜ}^{2}\left(\;\right)+{ℑ}^{2}\left(\;\right)}}\right|}^{0}$$

(35) $${\left.\frac{\partial \vartheta_{3}}{\partial {\tilde{\vartheta }}_{2}}\right|}^{0}={\left.\frac{-dl\cos{\tilde{\vartheta }}_{2}+l^{2}-l^{2}\cos\left({\tilde{\vartheta }}_{2}-{\tilde{\vartheta }}_{4}\right)}{{ℜ}^{2}\left(\;\right)+{ℑ}^{2}\left(\;\right)}\right|}^{0}$$

(36) $${\left.\frac{\partial \vartheta_{3}}{\partial {\tilde{\vartheta }}_{4}}\right|}^{0}={\left.\frac{dl\cos{\tilde{\vartheta }}_{4}+l^{2}-l^{2}\cos\left({\tilde{\vartheta }}_{2}-{\tilde{\vartheta }}_{4}\right)}{{ℜ}^{2}\left(\;\right)+{ℑ}^{2}\left(\;\right)}\right|}^{0}$$

Performing the Laplace transformation, so getting

$$\begin{array}{cc} \delta u(s) & ={\left.\frac{\partial \tilde{u}}{\partial {\tilde{\vartheta }}_{2}}\right|}^{0}\delta \vartheta_{2}\left(s\right)+{\left.\frac{\partial \tilde{u}}{\partial {\tilde{\vartheta }}_{4}}\right|}^{0}\delta \vartheta_{4}\left(s\right)\\ \delta \vartheta_{3}\left(s\right) & ={\left.\frac{\partial \vartheta_{3}}{\partial {\tilde{\vartheta }}_{2}}\right|}^{0}\delta \vartheta_{2}\left(s\right)+{\left.\frac{\partial \vartheta_{3}}{\partial {\tilde{\vartheta }}_{4}}\right|}^{0}\delta \vartheta_{4}\left(s\right) \end{array}$$

and substituting the obtained expressions in Equations (29) and (30), after some manipulations one gets

(37) $$\begin{array}{c} \left[I_{2}s^{2}+\left(cl\sin\left({\tilde{\vartheta }}_{2}^{0}-\vartheta_{3}^{0}\right){\left.\frac{\partial \tilde{u}}{\partial {\tilde{\vartheta }}_{2}}\right|}^{0}+c_{2}\right)s\right.+kl\sin\left({\tilde{\vartheta }}_{2}^{0}-\vartheta_{3}^{0}\right){\left.\frac{\partial \tilde{u}}{\partial {\tilde{\vartheta }}_{2}}\right|}^{0}+klu^{0}\cos\left({\tilde{\vartheta }}_{2}^{0}-\vartheta_{3}^{0}\right)+\\ \left.-klu^{0}\cos\left({\tilde{\vartheta }}_{2}^{0}-\vartheta_{3}^{0}\right){\left.\frac{\partial \vartheta_{3}}{\partial {\tilde{\vartheta }}_{2}}\right|}^{0}+k_{2}\right]\delta \vartheta_{2}\left(s\right)+\left[cl\sin\left({\tilde{\vartheta }}_{2}^{0}-\vartheta_{3}^{0}\right){\left.\frac{\partial \tilde{u}}{\partial {\tilde{\vartheta }}_{4}}\right|}^{0}s+\right.\\ \left.-klu^{0}\cos\left({\tilde{\vartheta }}_{2}^{0}-\vartheta_{3}^{0}\right){\left.\frac{\partial \vartheta_{3}}{\partial {\tilde{\vartheta }}_{4}}\right|}^{0}\right]\delta \vartheta_{4}\left(s\right)=\tau_{2}\left(s\right) \end{array}$$

for Equation (29), while Equation (30) becomes:

(38) $$\begin{array}{c} \left[I_{4}s^{2}+\left(cl\sin\left({\tilde{\vartheta }}_{4}^{0}-\vartheta_{3}^{0}\right){\left.\frac{\partial \tilde{u}}{\partial {\tilde{\vartheta }}_{4}}\right|}^{0}+c_{4}\right)s+\right.kl\sin\left({\tilde{\vartheta }}_{2}^{0}-\vartheta_{3}^{0}\right){\left.\frac{\partial \tilde{u}}{\partial {\tilde{\vartheta }}_{2}}\right|}^{0}+klu^{0}\cos\left({\tilde{\vartheta }}_{2}^{0}-\vartheta_{3}^{0}\right)+\\ \left.-klu^{0}\cos\left({\tilde{\vartheta }}_{2}^{0}-\vartheta_{3}^{0}\right){\left.\frac{\partial \vartheta_{3}}{\partial {\tilde{\vartheta }}_{2}}\right|}^{0}+k_{4}\right]\delta \vartheta_{4}\left(s\right)+\left[cl\sin\left({\tilde{\vartheta }}_{2}^{0}-\vartheta_{3}^{0}\right){\left.\frac{\partial \tilde{u}}{\partial {\tilde{\vartheta }}_{4}}\right|}^{0}s+\right.\\ \left.-klu^{0}\cos\left({\tilde{\vartheta }}_{2}^{0}-\vartheta_{3}^{0}\right){\left.\frac{\partial \vartheta_{3}}{\partial {\tilde{\vartheta }}_{4}}\right|}^{0}\right]\delta \vartheta_{2}\left(s\right)=0 \end{array}$$

Such expressions, under suitable definitions of coefficients $a_{i,j}$ and $b_{i,j}$, can be written as

$$\begin{array}{ccc} \left(a_{22}s^{2}+a_{21}s+a_{20}\right)\delta \vartheta_{2}\left(s\right)+\left(b_{21}s+b_{20}\right)\delta \vartheta_{4}\left(s\right) & = & \tau_{2}\left(s\right)\\ \left(a_{42}s^{2}+a_{41}s+a_{40}\right)\delta \vartheta_{4}\left(s\right)+\left(b_{41}s+b_{40}\right)\delta \vartheta_{2}\left(s\right) & = & 0 \end{array}$$

or, in a more compact form,

$$\left(\begin{array}{ccc} & a_{22}s^{2}+a_{21}s+a_{20} & b_{21}s+b_{20}\\ b_{41}s+b_{40} & a_{42}s^{2}+a_{41}s+a_{40} & \end{array}\right)\left(\begin{array}{c} \delta \vartheta_{2}\left(s\right)\\ \delta \vartheta_{4}\left(s\right) \end{array}\right)=\left(\begin{array}{c} \tau_{2}\left(s\right)\\ 0 \end{array}\right)$$

which, by inversion, gives

(39) $$\begin{array}{c} \left(\begin{array}{c} \delta \vartheta_{2}\left(s\right)\\ \delta \vartheta_{4}\left(s\right) \end{array}\right)={\left(\begin{array}{cc} a_{22}s^{2}+a_{21}s+a_{20} & b_{21}s+b_{20}\\ b_{41}s+b_{40} & a_{42}s^{2}+a_{41}s+a_{40} \end{array}\right)}^{-1}\left(\begin{array}{c} \tau_{2}\left(s\right)\\ 0 \end{array}\right)=\\ =\left(\begin{array}{cc} W_{1,1}\left(s\right) & W_{1,2}\left(s\right)\\ W_{2,1}\left(s\right) & W_{2,2}\left(s\right) \end{array}\right)\left(\begin{array}{c} \tau_{2}\left(s\right)\\ 0 \end{array}\right)=\left(\begin{array}{c} W_{1,1}\left(s\right)\\ W_{2,1}\left(s\right) \end{array}\right)\tau_{2}\left(s\right) \end{array}$$

The $\tau_{2}$–$\delta \vartheta_{4}$ relationship is then described by the transfer function $W_{2,1}\left(s\right)=\frac{\delta \vartheta_{4}\left(s\right)}{\tau_{2}\left(s\right)}$

(40) $$W_{2,1}\left(s\right)=\frac{N(s)}{D(s)}$$

where

(41) $$N\left(s\right)=-\left(b_{41}s+b_{40}\right)$$

and

(42) $$D\left(s\right)=\left(a_{22}s^{2}+a_{21}s+a_{20}\right)\left(a_{42}s^{2}+a_{41}s+a_{40}\right)-\left(b_{21}s+b_{20}\right)\left(b_{41}s+b_{40}\right)$$

Taking into consideration the linear representation of the relationship between the small action of the torque $\tau_{2}\left(t\right)$ and the corresponding small variation of angle $\delta \vartheta_{4}\left(t\right)$, which holds under the hypothesis previously discussed, the classical theory of the steady state response of a linear system under canonical inputs can be applied.

Within this framework, recalling that feeding an asymptotically stable linear system, described by a transfer function $W_{2,1}\left(s\right)$, with a sinusoidal input, which in this case can be set as

(43) $$\tau_{2}\left(t\right)=\varepsilon \sin\left(\bar{\omega }t\right),\;\bar{\omega }\in ℜ$$

the output, at steady state, assumes the form

(44) $$y_{ss}\left(t\right)=\varepsilon {\left.M(\omega )\right|}_{\omega =\bar{\omega }}\sin\left(\bar{\omega }t+{\left.\varphi (\omega )\right|}_{\omega =\bar{\omega }}\right)$$

where M(ω) and φ(ω) are such that

(45) $${\left.W(s)\right|}_{s=i\omega }=W\left(i\omega \right)=M\left(\omega \right)e^{i\varphi (\omega )}$$

**Figure 11:**
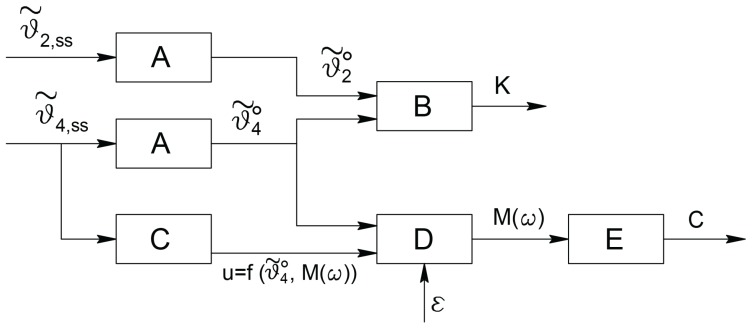
The complete control scheme for detecting the sample viscosity coefficient.

If the input torque is planned by the operator that performs the measurement, the constant value ε has to be fixed in order to guarantee that $∥\tau_{2}∥\ll \tau_{2}^{0}$; otherwise, if a closed loop control system as in Figure 11 is used, the input reference

(46) $${\tilde{\vartheta }}_{4,ref}={\tilde{\vartheta }}_{4}^{0}+\alpha \sin\left(\omega t\right)$$

must be chosen in order to produce, as gripper input, the torque (43), by setting the value of α suitably.

Then, it is possible to evaluate the unknown damping coefficient value c by measuring the amplitude of the steady state sinusoidal behaviour of the $\vartheta_{4}$ angle after that a sinusoidal exciting torque is applied to the first joint, thanks to the known dependency of M(ω) from the parameter c itself. A corresponding dependency holds for the phase displacement between input and output signals.

### 5.3. The Adopted Operational Scheme

The full measurement scheme is depicted in Figure 11.

With reference to Figure 11 and considering that noise cannot be neglected in real applications, Type A blocks are those whose outputs consist in the constant steady state values of the angles ${\tilde{\vartheta }}_{2}$ and ${\tilde{\vartheta }}_{4}$; noise reduction can be obtained designing A as an averaging filter or, somehow equivalently, as a low pass filter with a bandwidth $\Omega_{B}<\omega$. Type B block computes the inversion of the relationship between the amplitudes of the input and output constant values as a function of the elastic coefficient k. Such a relation is represented by the gain of the system under linearity hypothesis; in a more general case, it is a nonlinear function which can be analytically or experimentally computed.

The partial scheme represented in Figure 11 by Type A and B blocks allows the determination of the elastic coefficient k.

The remaining part is devoted to the computation of the damping coefficient c. The approach is the same as for the computation of k, but the operation must be performed under time variable behaviours of the signals involved. As previously introduced, inspired by the expressions in the linearly approximated case, the signals considered are sinusoidal. However, this kind of signals, can be conveniently used also when the dependency of the measured viscous coefficient on velocity is nonlinear. In fact, the use of different frequencies for the torque excitation affects directly the steady state velocities of all the mechanical components of the structure, including the sample tissue. Some of the following figures show how the value of c depends on the actual working frequency. With reference to Figure 11, the steady state behaviour of the ${\tilde{\vartheta }}_{4}$ angle is given by the constant value produced by the term ${\tilde{\vartheta }}_{4}^{0}$ in ${\tilde{\vartheta }}_{4,ref}$ plus the sinusoidal part as the response of the sinusoidal term $\alpha \sin\left(\omega t\right)$ in ${\tilde{\vartheta }}_{4,ref}$. Then, in order to extract the amplitude of the sinusoidal component only, which is the quantity related to the damping coefficient c, the scheme proposes the preliminary computation of the maximum value of the steady state response, performed by Type C block, and its comparison, performed by Type D block, with the constant component $\tilde{\vartheta_{4}^{0}}$ computed by block A.

Different choices can be adopted for Type C block according to the devices and the technologies used. A first possibility is to obtain the maximum value by means of a device which can detect directly such a quantity; then a *peak detector* or a *function envelope detector* can be used. They give the quantity εM(ω) plus the constant contribution. In these cases, block D has simply to subtract the constant term and to scale by the ε quantity to produce M(ω). A second possibility is represented by the computation of

(47) $$u=\int_{t_{i}}^{t_{i}+T}{\left({\tilde{\vartheta }}_{4,ss}\right)}^{2}dt$$

where $t_{i}$ is such that ${\tilde{\vartheta }}_{4,ss}\left(t_{i}\right)=0$ and ${\dot{\tilde{\vartheta }}}_{4,ss}\left(t_{i}\right)>0$.

From the expression of ${\tilde{\vartheta }}_{4,ss}={\tilde{\vartheta }}_{4}^{0}+\varepsilon M\left(\omega \right)\sin\left(\omega t+\phi (\omega )\right)$ Equation (47) gives

(48) $$\begin{array}{ccc} u & = & \int_{hT-T\frac{\phi (\omega )}{2\pi }}^{\left(h+1\right)T-T\frac{\phi (\omega )}{2\pi }}\left[{\tilde{\vartheta }}_{4}^{0}+\varepsilon M\left(\omega \right)\sin\left(\omega t+\phi (\omega )\right)\right]dt={\left(\tilde{\vartheta_{4}^{0}}\right)}^{2}+\frac{\varepsilon^{2}}{2}M\left(\omega \right) \end{array}$$

and in the block D the computation

(49) $$M\left(\omega \right)=\frac{1}{\varepsilon }\sqrt{2\left(u-{\left(\vartheta_{4}^{0}\right)}^{2}\right)}$$

has to be performed.

Analogously to Type B block, Type E block is devoted to the computation of the inverse function, analytically or experimentally determined, that relates the amplitude M(ω) to the damping coefficient c.

**Figure 12:**
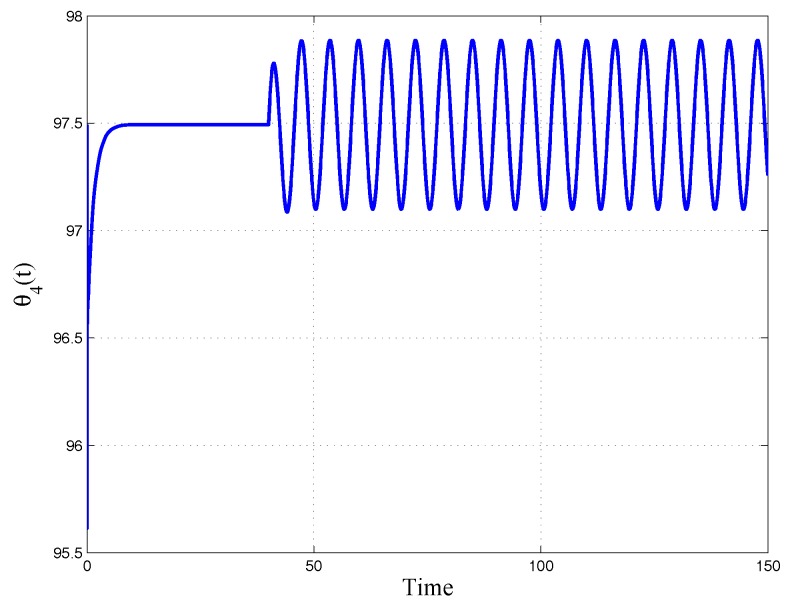
Generic time history for $\theta_{4}\left(t\right)$.

The generic output time evolution is reported in Figure 12, where the initial phase of the constant reference and the following effect of the sinusoidal input are well evidenced.

Numerical simulations, performed using Matlab^®^ and Simulink^®^ tools, can help to show the effectiveness of the proposed approach.

**Figure 13:**
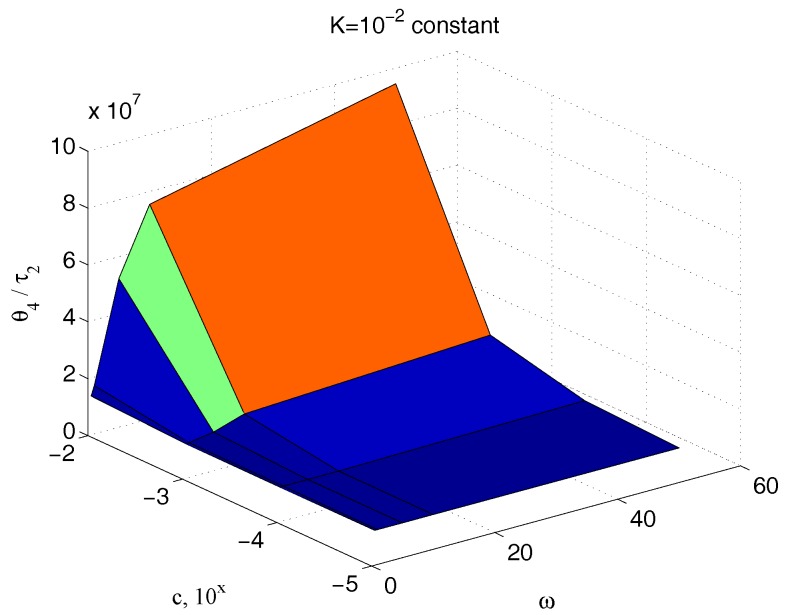
Dependency of $\vartheta_{4}/\tau_{2}$ with respect to c and ω for an assigned value of k.

**Figure 14:**
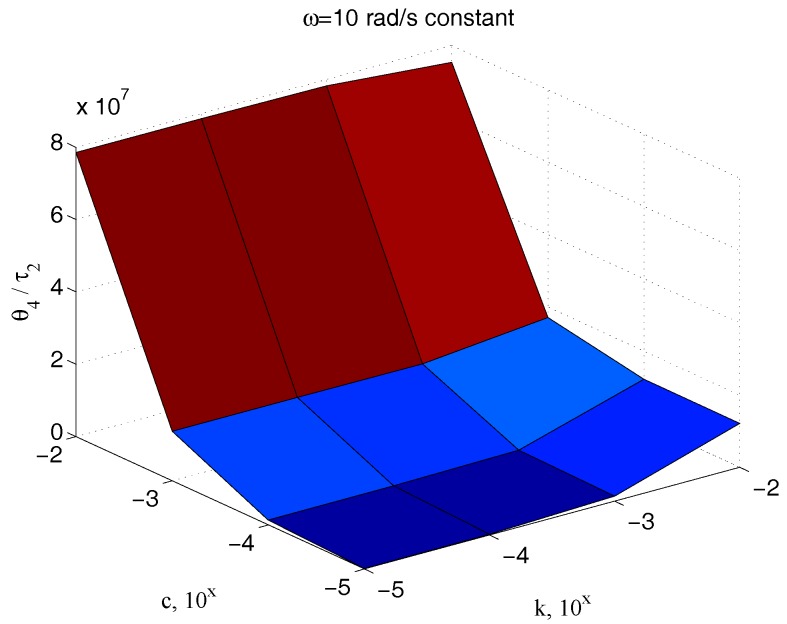
Dependency of $\vartheta_{4}/\tau_{2}$ with respect to c and k for an assigned value of ω.

Figure 13 reports the ratio of the amplitude of the sinusoidal output $\theta_{4}\left(t\right)$ to the corresponding input couple $\tau_{2}\left(t\right)$, for different values of the parameter c and for different frequencies, keeping the value of the elastic term k constant, $k=10^{-2}$. The great dependency of such a quantity from c at all the pulses greater than 5 rad/s confirms the effectiveness of the technique here proposed for its measurement. An equivalent result is obtained if the same ratio is depicted for different values of both the parameters c and k, for a fixed frequency. Figure 14 shows once again the high dependency of such a measure from c.

**Figure 15:**
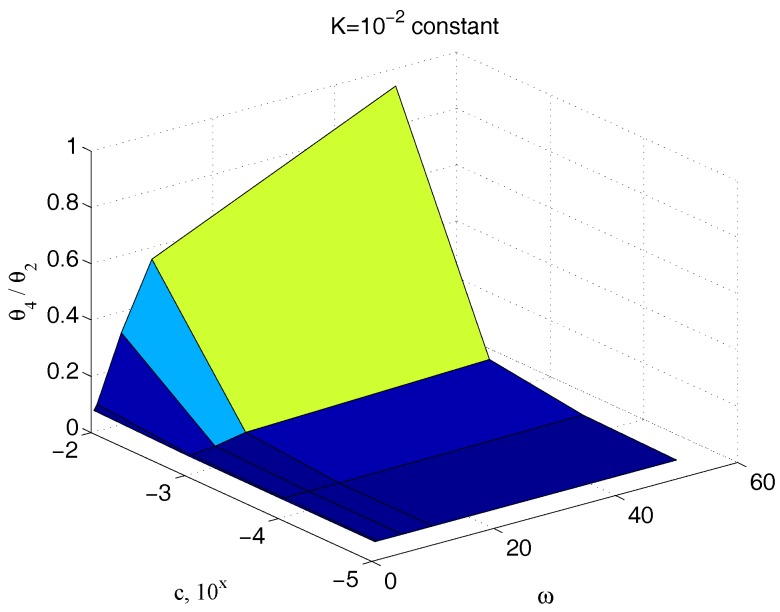
Dependency of $\vartheta_{4}/\vartheta_{2}$ with respect to c and ω for an assigned value of k.

**Figure 16:**
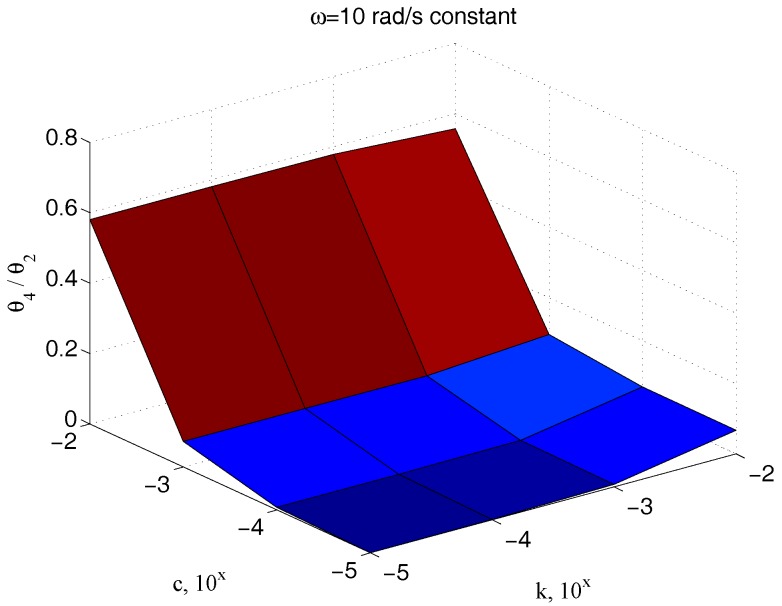
Dependency of $\vartheta_{4}/\vartheta_{2}$ with respect to c and k for an assigned value of ω.

The measurement of the input torque can be avoided, considering only angle measures. In this case, Figure 15 and Figure 16 have to be considered instead of Figure 13 and Figure 14, respectively. As expected, the results are substantially comparable, supporting this possible way as an easy alternative for the measurement scheme.

**Figure 17:**
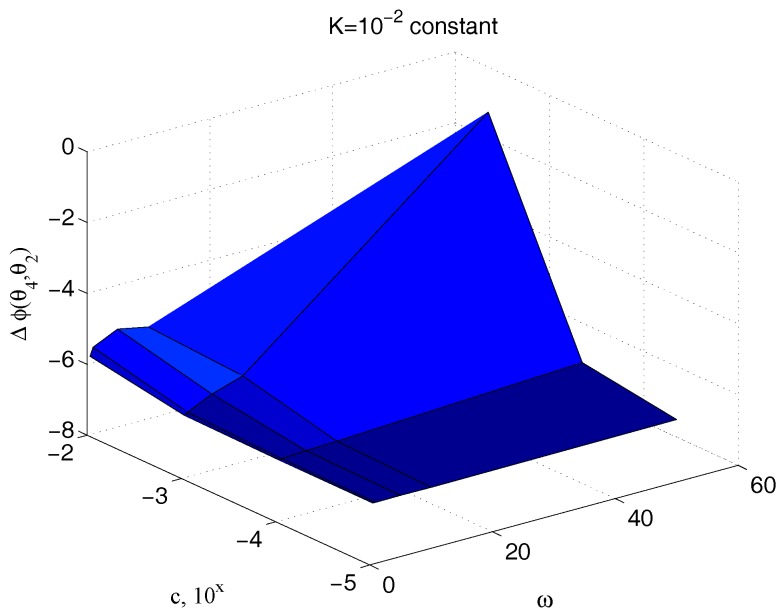
Dependency of the phase delay $\Delta \phi \left(\theta_{4},\theta_{2}\right)$ with respect to c and ω for an assigned value of k.

**Figure 18:**
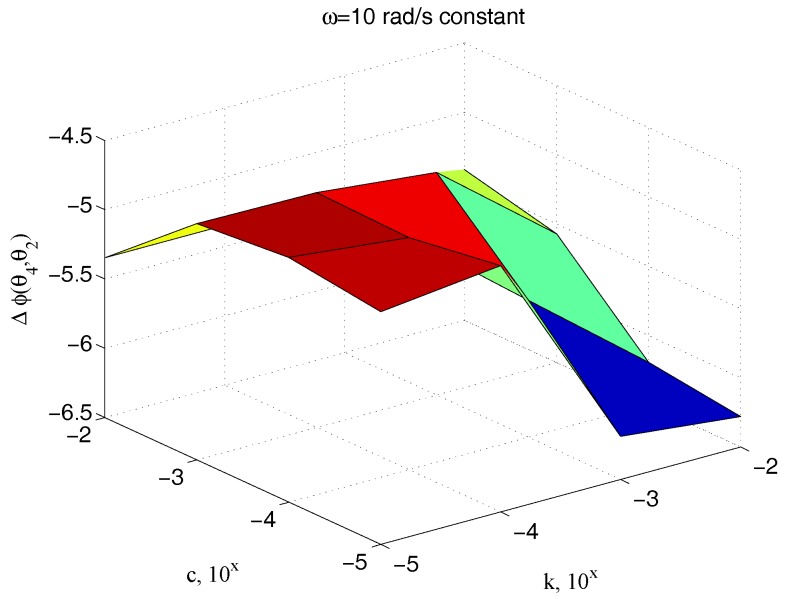
Dependency of the phase delay $\Delta \phi \left(\theta_{4},\theta_{2}\right)$ with respect to c and k for an assigned value of ω.

The relationships between input and output under sinusoidal signals involve also the phase delay $\Delta \phi \left(\theta_{4}\left(t\right),\theta_{2}\left(t\right)\right)$ between the two signals. Figure 17 illustrates the dependency of $\Delta \phi \left(\theta_{4}\left(t\right),\theta_{2}\left(t\right)\right)$ on the sample viscosity c and the signal angular frequency ω for an assigned value of the sample stiffness k, while Figure 18 shows how the phase delay depends on c and k, when the system is operated by the same pulse ω. The marked sensitivity of $\Delta \phi \left(\theta_{4}\left(t\right),\theta_{2}\left(t\right)\right)$ on c is confirmed.

## 6. Conclusions

The paper has shown the feasibility of a method to detect not only the stiffness but also the viscosity coefficient of a tissue sample for the purpose of identifying diseases in living organisms. All the presented simulations showed that the new microsystem has a great potential to enable researchers to investigate over samples having very limited size, about 200 μm × 200 μm. The new interdisciplinary and integrated approach revealed its high attitude to these kind of measurements mainly because it takes advantage from different fields, such as mechanics, modern micro–fabrication techniques and control theory. The practical utility of the proposed approach will soon be proven by carrying out an experimental campaign on real biological tissues.

## Figures and Tables

**Table 1:** Parameter values given in input to the developed model.

Parameter	Value	Parameter	Value
${\hat{\vartheta }}_{2}$	1.44 rad	${\hat{R}}_{a}$	$7.78\times 10^{-4}$ m
${\hat{\vartheta }}_{4}$	1.70 rad	b	$5\times 10^{-6}$ m
β	4.20 rad	h	$40\times 10^{-6}$ m
${\hat{\vartheta }}_{3}$	0 rad	$J_{p\;2}$, $J_{p\;4}$	$1.34\times 10^{-13}$ m${}^{4}$
$\hat{u}$	$150\times 10^{-6}$ m	$m_{2}$, $m_{4}$	$1.9\times 10^{-8}$ kg
d	$5.47\times 10^{-4}$ m	$I_{2}$, $I_{4}$	$1.25\times 10^{-14}$ kg·m${}^{2}$
l	$1.496\times 10^{-3}$ m	μ	$18.6\times 10^{-6}$ kg/m·s
$r_{b}$	$62.5\times 10^{-6}$ m	$k_{2}$, $k_{4}$	$0.30\times 10^{-6}$ kg·m${}^{2}$/$s^{2}$·rad
$z_{0}$	2 $\times 10^{-6}$ m	$c_{2}$, $c_{4}$	$1.24\times 10^{-12}$ kg·m${}^{2}$/s·rad
